# The Early Treatment and Diagnosis of Anterior Poliomyelitis
*Read at Bath, at a Meeting of the Bath and Bristol Branch of the British Medical Association, November 27th, 1929.


**Published:** 1929

**Authors:** R. G. Gordon

**Affiliations:** Physician to the Bath, Somerset, Wilts and Dorset Orthopædic Hospital


### THE EARLY TREATMENT AND DIAGNOSIS OF ANTERIOR POLIOMYELITIS.*

BY

R. G. GORDON, M.D., D.Sc., F.R.C.P.E.,

*Physician to the Bath, Somerset, Wilts and Dorset Orthopœdic Hospital.*

THE occurrence of a widespread epidemic of poliomyelitis in this district has prompted me to draw attention to certain aspects of the disease. So far as my personal experience goes, through observation of the cases admitted to the Bath Orthopædic Hospital, the present epidemic began somewhere about the beginning of August and covers an area from Bridport to Swindon. It does not appear that the cases are exceedingly numerous, and, as a rule, this feature has been found in all the epidemics which have been studied, namely a wide epidemic area with comparatively few cases, the incidence confined for the most part to children, though a few adults are frequently affected, at any rate in the more severe epidemics. Sometimes, however, this is not so. For example, a few years ago an epidemic occurred in the small Devonshire village of Stoke Rivers, when out of a population of 119 not less than 45 individuals were affected, all types of the disease being met with. As a contrast to this, there was an epidemic in Edinburgh

* Read at Bath, at a Meeting of the Bath and Bristol Branch of the British Medical Association, November 27th, 1929.

about the same time in which only 62 cases occurred out of a population of 420,000, and all cases were of the spinal type.

The mortality rates are relatively more severe the older the patient, though this varies from epidemic to epidemic. Incidentally, it is not always realized how fatal a disease poliomyelitis may be, yet the official figures for England and Wales for 1st July, 1925, to 30th June, 1926, were 264 cases notified and 136 deaths—over 50 per cent. In any epidemic it is noticeable how cases vary in severity, and how many cases seem to be quite abortive, passing off entirely after a few initial symptoms. A consideration of the widespread area of incidence, the variability in severity of cases, and the relative immunity of adults, but the greater fatality of the disease amongst those adults who do not enjoy this immunity, has led most observers to postulate the theory that the virus is very widespread, but is only of pathological significance to a few individuals, the rest contracting the disease in what is called a subclinical form, that is, without the exhibition of symptoms. These observers further believe that in this subclinical form the person harbouring it is highly infectious, and that such people may act as carriers. The power to carry the disease, however, only seems to last for about three weeks; but chains of carriers may keep the infection alive, and thus account for sporadic cases which occur continuously in almost all districts.

Work by American authorities in the New England States has shown that epidemics seem to obey Commander Dudley's laws. Dudley showed in experiments on rats and mice with epidemic infections that if colonies are kept strictly segregated the virulence of the infecting virus tends to diminish and the colony acquires immunity relatively quickly. If, however, new individuals are introduced into the community and acquire the disease, the virulence of the virus is increased, so that the members originally immune may succumb to the disease. This is important in relation to epidemics in boarding-schools, for the really proper procedure would be to segregate the whole school, though the headmaster who would have the courage to do this might be far to seek. However, he would be right, for the carrier of the infection is less likely to be the child actually suffering from the disease, whose infectivity does not last longer than three weeks, while the incubation period of nervous symptoms is from two to twelve days, than an apparently healthy child who would naturally mix freely with others on his return home. The nature of the virus concerned is not known, but that it is of an organismal nature is shown by the fact that the disease may be conveyed to monkeys, specially *macacus rhesus*. This can be done readily by intracerebral injection, but not easily by subcutaneous injections. Active virus can be obtained from the secretions and also from the peripheral nerves of monkeys dead of the disease. In human cases actual contact does not seem to be potent in spreading poliomyelitis, but it would appear to be conveyed by means of secretions, specially by nasal secretions and diarrhœa.

The virus may lodge, therefore, in the upper respiratory passages, the tonsils and fauces and in the gastro-intestinal tract, and would seem to spread from these areas to the central nervous system by way of the perineural lymphatics along the course of the peripheral nerves.

During an epidemic all secretions and excretions of the nose, throat or bowel must be destroyed and care be taken of droplet contact, while those associated with the case should use disinfectant sprays and gargles.

The onset of the disease is usually sudden, but may be gradual, and the typical course is by no means always followed. Walshe[1] has described three stages in the course of development of the illness. First, there is a phase of general invasion, when the virus may be found in the liver, spleen, lymphatic glands, and (at any rate in experimental animals) in the bone marrow. During this stage the patient may exhibit symptoms of coryza, tonsillitis, or gastro-intestinal disturbance with diarrhœa and vomiting.

Next, the virus invades the subarachnoid space, and the patient exhibits headache, pain in the limbs and back, is frequently fretful and cries a great deal. Sometimes he is apathetic and many even pass into coma, rarely there is delirium and convulsions. At this stage the spine is frequently resistant to passive flexion—the spine sign of the Americans. During this stage if the cerebro-spinal fluid is drawn off and examined the cells are found to be increased from 20 to 2,000 per c.cm. and to include polymorphs ; the globulin is raised, but the sugar and chlorides remain normal. Cultures are always sterile. Incidentally, the drawing off of spinal fluid, which may be under some tension, often relieves symptoms markedly.

This meningeal stage may follow the general stage rapidly, but there is sometimes a pause of one or two days during which the patient is supposed to have completely recovered from a passing and trivial malady.

Occasionally these two stages may be absent or show such slight manifestations as to escape notice altogether, and the patient passes straight into the third stage when the virus attacks some part of the neuraxis. In cases ending fatally in the early stages the reaction to the invasion of the virus is seen to be an intense hyperæmia and engorgement of the blood-vessels, and many of the nervous elements are compressed out of action, so to speak, by the resulting pressure and œdema, but recover when this is removed. One reason why the anterior horn region of the cord is so frequently affected is that it enjoys a specially rich blood supply.

The old conception implied by the cognomen of "anterior poliomyelitis" must be abandoned, for the disease takes many forms, the classification usually adopted being that of Wickman modified by Collier[2] :—

1. The subclinical type, in which the patient exhibits no symptoms but is a carrier of the disease. He is generally himself immune, but sometimes he succumbs.

2. The abortive case, who exhibits pyrexia, headache, signs of meningeal irritation—perhaps nystagmus, jerks of the limbs and some loss of reflex action, superficial or deep, and occasionally some loss of power, but in a few days completely recovers.

3. The spinal case, with involvement of the anterior horn cells. Note that pain is almost always present at the beginning, and that the muscles involved may remain tender for some time. Tenderness of the muscles is regarded as a better prognostic sign than complete muscular anæsthesia. Skin anæsthesias may also occur. Cases which retain their reflexes in spite of widespread paralysis will probably make a complete recovery, though loss of reflexes in the early stages does not mean a necessarily bad prognosis.

A transverse myelitis may occur with spastic paralysis below the lesion, with paralysis of the bladder reflexes. As a rule these cases recover, but not always.

4. A so-called neuritic type, where pain and tenderness of muscles is very marked. It must be remembered that pain from other causes usually increases tendon reflexes, while poliomyelitis abolishes them. Such cases often develop early contractures, especially as the limbs cannot be stretched and fixed because of the pain.

5. The spreading type. Sometimes, though rarely, the paralysis spreads either upwards or downwards, as in the Landry syndrome, and these are the cases that tend to be fatal as the respiratory muscles become involved.

6. The meningeal type. Abortive cases with meningeal symptoms are usually classed in this group, but there are a few who go on to simulate a tuberculous meningitis.

7. A cerebellar type with marked ataxy and nystagmus.

8. The so-called brain-stem type, with involvement of cranial nerves is probably encephalitis lethargica and not poliomyelitis.

9. The cerebral type, with convulsions followed by hemiplegia or other cerebral defect.

10. Relapsing cases occasionally occur, but the relapses, though disquieting, are not usually serious.

11. Mixed types are very rare indeed, though they do occur.

Before passing on to the question of treatment, I would like to draw attention to some valuable observations carried out by Dr. Whidbourne while she was house physician at the Bath Orthopædic Hospital.

She made a careful inquiry into the history of onset of 100 cases admitted into the hospital, with the following results :—

She found that 17 gave a history of an insidious onset, that is to say that no acute symptoms were observed or remembered.

Eleven were said to have shown a sudden onset of paralysis without any preliminary symptoms being observed. In 9 cases the onset was definitely associated in the minds of the parents with trauma, while in 61 initial symptoms of one sort or another were recorded. Of these 18 were described as vague, 22 as moderate and 21 as severe.

Of initial symptoms of the general group, apart from paralysis, fever was present in 38, vomiting in 17, diarrhœa in 1, marked constipation in 6. Coryza was noted in 6 cases.

Of the stage of arachnoid involvement symptoms were observed as follows : pain and tenderness in 38, headache in 33, head retraction in 13, difficulty in micturition in 9, delirium in 6, convulsions in 6, giddiness in 4, and unusual sleepiness in 2. The disturbance of bladder reflex is a matter of some interest, as this is not usually associated with poliomyelitis.

*Treatment.*—Since the diagnosis of poliomyelitis is very difficult, if not impossible, before the paralytic stage, except in the most definite epidemics, the measures for aborting the disease cannot usually be employed, since they are of little or no use once the neuraxis has become involved. Of drugs, hexamine has been recommended by Flexner and others, since formaldehyde is found to pass into the cerebro-spinal fluid. In ordinary doses it seems to be of very little use, but it may be more useful in massive doses of 1–2 drachms daily with alkalies. The serum of convalescent patients given intrathecally, intravenously or even subcutaneously has been found useful if given early in 10–20 cc. doses, but this is often hard to obtain, unless there is a severe and relatively circumscribed epidemic.

Practically, then, we are reduced to the treatment of the early paralytic cases. Lumbar puncture is often useful, not only for establishing the diagnosis, but also to remove symptoms, especially pain and restlessness. This is of great importance, for during the early period the one requisite first, last and all the time is rest, absolute rest and continuous rest. Absolutely no unnecessary movement should be allowed, and great care should be taken that a proper posture of head, trunk and limbs is maintained all the time. I have no hesitation whatever in saying that this cannot be obtained in an ordinary bed, and in my opinion every case which is not obviously of the abortive type should be nursed on a frame for at least two months from the initial symptoms, and longer if there is any sign of tenderness of the muscles remaining. Then the patient may be taken off the frame for a short time daily, during which careful massage may be given to the weak muscles. The masseuse must observe very carefully that real progress is being made, any retrogression of the muscle being an indication for immediate cessation of treatment and restoration of rest for a further ten days or a fortnight. Too often the zealous masseuse thinks that the weakening of the muscle means insufficient work on her part, and, redoubling her efforts, destroys for ever the chance of recovery of the muscle. Electricity should never be administered until a comparatively late stage in the treatment. Above everything, a weak muscle must never be allowed to stretch even for five minutes. The relatively powerful antagonist muscle is, as it were, always on the look-out for a chance to assert itself, and that way lie contractures, deformities and infinite difficulty in restoring muscle balance. It is not too much to say that if care were taken in the early stages of poliomyelitis the incomes of orthopædic surgeons would be halved, and if that would seriously decrease the national receipts from income tax it would save much maintenance in orthopædic hospitals.

Now it may be asked, Why all this insistence on absolute rest in early stages—no movement, no massage, no electricity? Would it not be better to train the weak muscle to get back its strength from the very beginning? This might be so if it were not for the existence of a muscle sense; but we must remember that every movement, every manipulation and every electric stimulus sends a message up the afferent nerve, that this travels to the posterior ganglion, from there to the spinal grey matter and, redistributed here, a stimulus proceeds to the anterior horn cells governing the motor nerves to the muscle concerned, for it is thus that muscular tone is maintained under the direction of higher centres. So we see that whatever we do to the affected muscle we cannot help disturbing the anterior horn cell which is the seat of inflammation and which must at all costs be kept at rest. So I say again that, except when it is impossible on account of pain, which can usually be relieved by lumbar puncture, rest in a frame is the only and the essential treatment, once paralysis has become manifest, for at least two months. This may seem a long time and a hard discipline in apparently mild cases, but what is two months on his back for a child, compared to a life of crippledom ?

REFERENCES.

[1] *Lancet,* 1927, i., p. 326.

[2] *Loc. cit.,* p. 321.

